# Health Insurance Notes

**Published:** 1923-06

**Authors:** 


### NATIONAL HEALTH INSURANCE NOTES.

### INSTRUCTING THE INSURED.

It is of importance for the smooth working of Medical Benefit that every insured person should have clearly in his mind such fundamental things as that he must be in possession of a medical card, how to get a fresh one if he loses his card, how to obtain treatment when away from home, or in an emergency, and the simple rules about certificates. The Leicestershire Insurance Committee has issued a large notice containing information on these and other points, and the doctors have been asked to hang the notices up prominently in their waiting-rooms. It is in the doctors' interest to do this, and the plan might be adopted everywhere with advantage.

### A WORD FOR THE VILLAGE DOCTOR.

Dr. Williams-Freeman, of Hampshire, the protagonist of the rural practitioners, contributes an interesting article to a recent issue of the *British Medical Journal*. He writes of the increasing difficulty of the country doctor, who must live on a certain social level or his best private patients will fall away; who must keep up to date intellectually and professionally, and must necessarily be ready to give a wider range of service than his town brethren, who have hospital facilities to fall back upon; and who must educate his children well. The good man, says Dr. Williams-Freeman, will not go into country practice, unless he has private means; and yet the good type of village practitioner is worth keeping. So far as insurance patients are concerned he must do, and does, a good deal outside the letter of his contract. Dr. Freeman expresses himself as satisfied with the aggregate amount of the present mileage grant, but says that it is unequally distributed. This he recognises to be a matter for his professional brethren to put right. As to his further plea for special grants from Insurance funds for really necessitous areas, such as have been made in Scotland from Exchequer grants in the past, there will always, we believe, be sympathetic consideration and effective help when the facts of particular cases are properly established.

### DISPENSING BY CHEMISTS.

We find some difficulty in understanding why the *National Insurance Gazette* from time to time tilts at the chemists as a body. It is just as unreasonable as tilting at the panel doctors in the mass. Where there are defaulting chemists, by all means let them be penalised, and, in suitable cases, removed from the panel. But there are perfectly good reasons behind the National Insurance provisions for separating the dispensing and the prescribing, and it would surely be a retrograde step to hand the dispensing back to the doctors and to give them a discretion—so we understand the *Gazette* to suggest—to arrange with the chemists to dispense.

### AN EXCELLENT SPIRIT.

The Joint Committee appointed by the Guildhall Conference have shown in their first meetings the excellent spirit which marked the Guildhall proceedings. The unanimous appointment of Dr. Brackenbury as Chairman, on Sir Thomas Neill's suggestion, was a graceful act on the part of the Society representatives. Mr. Gill Hodgson will make a first-rate Secretary. Some useful work will undoubtedly result, and we look forward especially to the loud requests for reform of the panel system being circumscribed and defined.

### SURGERY ACCOMMODATION.

From the first meeting of the Joint Committee issued the sound recommendation that questions of whether surgery and waiting-room accommodation is adequate should be jointly investigated by the Insurance Committee and the Panel Committee. In practice, when a visit to a doctor's surgery is made, two representatives, one from each body, should be adequate and more effective than any larger number. An interesting suggestion has recently been made by Sir Thomas Neill that some of the doctors in congested areas like the East-End of London might arrange with the hospital authorities for the use of a suite of rooms in the hospital buildings, where they could see their own patients under better conditions and with better equipment than in their own surgeries. This would also tend to better co-operation between the panel doctor and the hospitals.

### OUR HELPFUL CONTEMPORARIES.

The following suggestions in two of our contemporaries do not, we fear, carry us far along the road of reform: 1. "The Ministry of Health has an opportunity of justifying its existence in the proposed reorganisation of the panel system. If it likes, it can now organise an A1 Health Service—which should have been done long ago."—(*John Bull.*) 2. "Largely as a consequence of the panel system a sort of general practitioner exists who is profoundly incompetent and who is a positive danger to the community. . . A very good plan is that already being adopted by working people—to pay the tax because they have to, but to ignore the panel doctor, and go to the hospitals, where the treatment has improved since they have had to pay."—(*New Witness.*)

---

**Sanitary Inspectors' Association.**

The Sanitary Inspectors' Association will hold its Annual Conference at Rhyl, from September 4 to 7. The arrangements for the Conference will include the annual general meeting and members' conference, a conference lecture on various phases of public health work, an official reception, the President's inaugural address, and papers on sanitary progress in Rhyl.

